# Comparison of Tamsulosin and Tadalafil effects in LUTS treatment considering patients' atherosclerosis risk level

**DOI:** 10.1016/j.amsu.2022.104137

**Published:** 2022-07-09

**Authors:** Ali Tavoosian, Leonardo Oliveira Reis, Pavan Aluru, Alireza Khajavi, Seyed Mohammad Kazem Aghamir

**Affiliations:** a Urology Research Center, Tehran University of Medical Sciences, Tehran, Iran; b UroScience and Department of Surgery (Urology), School of Medical Sciences, University of Campinas, Unicamp, Pontifical Catholic University of Campinas, PUC-Campinas, Campinas, São Paulo, Brazil; c Department of Urology, Sunderland Royal Hospital, UK; d Faculty of Paramedical Sciences, Shahid Beheshti University of Medical Sciences, Tehran, Iran

**Keywords:** Lower urinary tract symptoms, Benign prostatic hyperplasia, Tadalafil, Tamsulosin, Treatment

## Abstract

**Introduction:**

To date, no study evaluates the effect of atherosclerosis risk level on the efficacy of BPH drug therapies. Therefore, the present study aimed to assess the effect of atherosclerosis risk levels on the effectiveness of Tamsulosin and Tadalafil in LUTS treatment.

**Methods:**

The present study was a randomized clinical trial that assessed men with LUTS symptoms (at least six months). The inclusion criteria were being older than 50 years, international prostate symptom score (IPSS) ≥ 13, and maximum urinary flow rate (Qmax) between 4 and 15 ml/s. Framingham Risk Score was used to measure atherosclerosis risk. The patients were classified into four groups, including group 1: Patients with low risk and treated with Tamsulosin (0.4 mg/day), group 2: Patients with low risk and treated with Tadalafil (5 mg/day), group 3: Patients with high risk and treated with Tamsulosin (0.4 mg/day), group 4: Patients with high risk and treated with Tadalafil (5 mg/day).

**Results:**

The study included 44 and 38 patients receiving Tamsulosin and Tadalafil, respectively. The means (SD) of the baseline age for the Tamsulosin and Tadalafil groups were equal to 60.6 (6.8) and 58.8 (6.7), respectively (p-value = 0.213). The models revealed no impact of the atherosclerosis risk level on the drugs' effects (p-values = 0.378, 0.975, 0.743 for IPSS, QMAX, and VOID, respectively).

**Conclusions:**

The present study's findings could not show the impact of atherosclerosis risk levels on the efficiency of Tamsolusin and Tadalafil in men with LUTS.

## Highlights

• Assessing the impact of atherosclerosis risk level on the effectiveness of Tamsulosin and Tadalafil in LUTS treatment.
• The present study's findings could not show the impact of atherosclerosis risk level on the efficiency of Tamsulosin and Tadalafil in men with LUTS.

## 1 Introduction

Benign prostatic hyperplasia (BPH) is approximately common in aging men, surpassing 80% in those over 80 years [1]. Lower urinary tract symptoms (LUTS) happen due to bladder outlet obstruction and increases in muscle tone. LUTS prevalence increases with age, and documents show that more than half of men over 50 years have LUTS [2,3]. LUTS is correlated with impaired bladder emptying or storage. Voiding signs often display initially and might include urinary delay, a limited urinary stream, and the feeling of incomplete bladder emptying. Storage symptoms include urinary frequency, nocturia, urinary urgency, and urge incontinence, which might happen late in the course and are associated with detrusor hypertrophy and irritability [4]. LUTS is not associated with significant morbidity or mortality but can negatively impair the quality of life [5,6].

BPH has various treatment protocols, such as surgical or pharmacological treatment. Surgical treatment of BPH is not without complications; therefore, many patients try to relieve urinary symptoms with drug medication. The standard pharmacological treatment for men with moderate to severe urinary symptoms includes α1- Adrenergic receptor antagonist and 5α-Reductase inhibitor [7]. Various agents can affect the effectiveness of drugs in treating LUTS; the document suggests atherosclerosis might influence the efficacy of the drugs. Azadzoi et al. assessed the effect of chronic ischemia on prostatic smooth muscle contraction in the rabbit. They showed that Doxazosin administration in atherosclerosis groups had less therapeutic effects than in the control group [8].

Tadalafil is a phosphodiesterase-5 (PDE5-I) compound that improves blood circulation, and it is effective in men with erectile dysfunction (ED), which might also have a role in the treatment of LUTS [[9], [10], [11]]. The biological mechanisms of PDE5-I in LUTS improvement have not been fully known. PDE5-I drugs may improve pelvic blood flow through increased vascular perfusion in the prostate and bladder neck [12]. To date, no study evaluates the effect of atherosclerosis risk level on the efficacy of BPH drug therapies. Therefore, the present study aimed to assess the effect of atherosclerosis risk level on the effectiveness of Tamsulosin and Tadalafil in LUTS treatment.

## 2 Methods

The present study was a randomized clinical trial study that the Ethics Committee of Tehran University of Medical Sciences approved the study *(IR.TUMS.SINAHOSPITAL.REC.1399.042,* https://www.irct.ir/trial/44611***).* Moreover, the study has been reported in line with the CONSORT criteria** [13]**.**

Men with LUTS symptoms (at least six months) participated in the study. The inclusion criteria were being older than 50 years, international prostate symptom score (IPSS) ≥ 13, and maximum urinary flow rate (Qmax) between 4 and 15 ml/s.

Exclusion criteria were PSA of more than 10, residual urine ≥300 ml, Finasteride intake during the last three months, anti-androgen drugs intake, history of surgery or pelvic radiotherapy, malignancy and trauma history of the lower urinary tract, urinary retention, bladder stone, urinary tract infection, urethral stricture, a neurological disease affecting bladder function, prostate cancer, secondary obstruction of prostate median lobe, liver or kidney failure, cardiovascular diseases, treatment with nitrates, as well as uncontrolled diabetes (HbA1c > 9%).

All participants completed the written informed consent after explaining the study's aim. The Framingham Risk Score was used to measure atherosclerosis risk, and the score obtained relates to the chance of cardiovascular diseases in the next ten years. Low risk was defined as less than 20%, and the high risk as more than 20% [14]. So, the patients were classified into four groups:

1. Patients with low risk and treated with Tamsulosin (0.4 mg/day)
2. Patients with low risk and treated with Tadalafil (5 mg/day)
3. Patients with high risk and treated with Tamsulosin (0.4 mg/day)
4. Patients with high risk and treated with Tadalafil (5 mg/day)

If the patients took alpha-blocker drugs before entering the study, they would have entered a two-week wash-out period. Post-void residual volume (PVR), IPSS, IPSS-QOL, and international index of erectile function (IIEF) were evaluated before treatment and 4, 8, and 12 weeks after initiation of treatment. Qmax was assessed at the first and end of the study.

## 3 Statistical analysis

The continuous variables are compared between two drugs using either a *t*-test or Mann-Whitney test, depending on the variable's distribution to follow the Normal distribution. The Chi-squared test is used to compare the discrete variables between two drugs. Moreover, the longitudinal models were fitted to the response variables to assess the impact of the atherosclerosis risk on the drugs' effects.

## 4 Results

The study included 44 and 38 patients receiving Tamsulosin and Tadalafil, respectively. The means (SD) of the baseline age for the Tamsulosin and Tadalafil groups were equal to 60.6 (6.8) and 58.8 (6.7), respectively (p-value = 0.213). There was no missing in the measurements. A description of the covariates is presented in Table 1, separated into groups.

**Table 1:** Comparison of the covariates between Tamsulosin and Tadalafil.

Covariates		Group		p-value
		Tamsulosin	Tadalafil	
PSA, median (IQR)		1.8 (0.9,2.8)	1.1 (0.6–1.7)	0.006^a^
SBP, mean (SD)		134.3 (21.1)	127.6 (16.4)	0.117^b^
HDL, mean (SD)		40.5 (6.8)	40.8 (7.9)	0.846^b^
Cholesterol, mean (SD)		157.8 (56.4)	158.5 (62.2)	0.959^b^
Framingham score, median (IQR)		6 (3–11)	5 (3–11)	0.436^a^
Risk, number (%)	Low	25 (56.8%)	25 (65.8%)	0.406^c^
	High	19 (43.2%)	13 (34.2%)	
DM (yes), number (%)		16 (36.4%)	14 (36.8%)	0.964^c^
Smoking (yes), number (%)		15 (34.1%)	17 (44.7%)	0.324^c^
Opium (yes), number (%)		6 (13.6%)	7 (18.4%)	0.554^c^

a Mann-Whitney test.

b *t*-test.

c Chi-squared test.

**Fig. 1:**
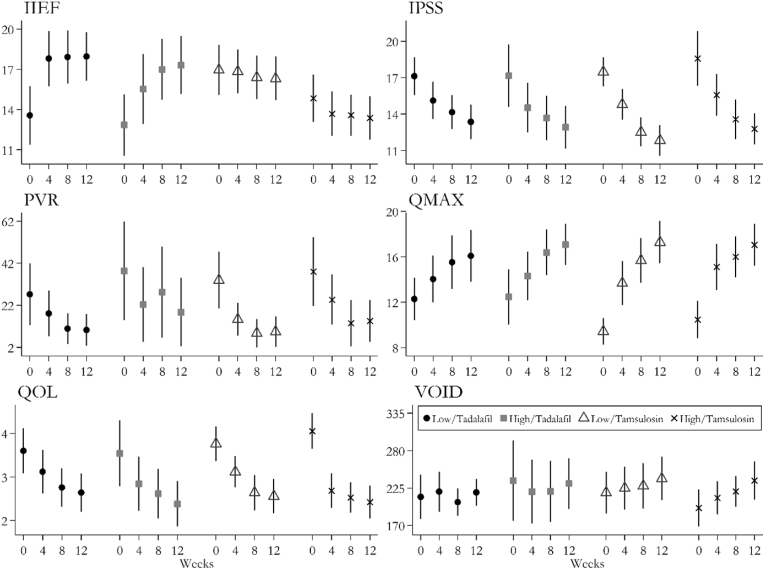
The response variables' trends over the measurements of baseline and weeks 4, 8, and 12.

Moreover, the response variables’ trends over the measurements of baseline and weeks 4, 8, and 12 are presented in Fig. 1, using means and 95% confidence intervals, comparing the drugs and risks.

Finally, as the IPSS and QMAX response variables followed the Normal distribution, the longitudinal models were fitted. The models revealed no impact of the atherosclerosis risk level on the drugs' effects (p-values = 0.378 and 0.975 for IPSS and QMAX, respectively). Besides applying the reverse transformation to the non-normal response of VOID, the changed response was put in the longitudinal model, and once more, no impact of the risk level on the drugs’ effects was observed (p-value = 0.743).

## 5 Discussion

In the present study, we assessed the effect of atherosclerosis risk levels on the effectiveness of Tamsulosin and Tadalafil in LUTS treatment and the findings showed that atherosclerosis risk levels have no impact on the efficiency of Tamsolusin and Tadalafil in men with LUTS and in patients with atherosclerosis, the general treatment protocol could be used.

In recent years, pharmacological treatments for LUTS have received much attention. Numerous studies assessed the Tamsulosin and Tadalafil for LUTS, and the findings of studies are inconsistent in some aspects. Several studies have not described improvement in Qmax or PVR with tadalafil compared with placebo, although some development was indicated for the IPSS [[14], [15], [16]]. Similarly, the tamsulosin and tadalafil combination treatment did not increase Qmax or PVR compared with the tamsulosin-only treatment [17]. **Oelke et al. performed the randomized, parallel, placebo-controlled clinical trial and assessed the monotherapy with tadalafil or tamsulosin on LUTS in men. Patients finished a 4-wk placebo run-in before randomization to placebo (n=172), tamsulosin 0.4 mg (n=168), or tadalafil 5 mg (n=171), once daily for 12 wk** the findings of the study showed that monotherapy with tadalafil or tamsulosin significantly improved IPSS and Qmax similar placebo group [18].

The document recommends that Tadalafil, which revolutionized the treatment of erectile dysfunction (ED), might also have a role in treating LUTS [19]. The PDE-5 inhibitors may develop LUTS through different biological mechanisms, including Rho-associated protein kinase (Rk) deactivation, reductions in pelvic atherosclerosis, and alterations in nitric oxide (NO) [20]. These hypotheses do not happen separately and share characteristics leading to upregulation of messengers affecting relaxation and contraction of tissue beds in the urogenital tract. PDE-5 might help prevent pelvic atherosclerosis seen in patients with LUTS. Azadzoi et al. assessed the effect of chronic ischemia on prostatic smooth muscle contraction in the rabbit. They found that ischemia causes obvious structural impairment and increases prostatic smooth muscle contraction. Doxazosin significantly increased cGMP release in the control group but not in the ischemic group. In addition, it is observed that stimulators of cGMP synthesis and NO production improved the effectiveness of doxazosin in declining prostatic tissue contraction [8]. To date, no study evaluates pelvic atherosclerosis status's effect on alpha-blockers' efficacy, especially phosphodiesterase inhibitors drugs in males with LUTS. The present study assessed the effect of atherosclerosis on the effectiveness of Tamsulosin and Tadalafil in LUTS treatment. The present study's findings revealed no impact of the atherosclerosis risk level on the drugs' effects, and there were no significant differences in IPSS, Qol, PVR, and Qmax between groups. So, Tamsulosin and Tadalafil had a similar effect in treating LUTS. This study's most important limiting factor was the low number of patients in the high-risk group treated with Tadalafil. It was suggested that future studies could be performed in larger sample size.

## 6 Conclusion

The findings of the present study could not show the impact of atherosclerosis risk levels on the efficiency of Tamsolusin and Tadalafil in men with LUTS.

## Ethical approval

The present study was the randomized clinical trial study run under the Ethics Committee of Tehran University of Medical Sciences supervision *(IR.TUMS.SINAHOSPITAL.REC.1399.042)* and approved by the Iranian Registry of Clinical Trials *(IRCT20191230045943N1).* Patients enter the study after signing the written informed consent.

## Sources of funding

No funding.

## Funding

There is no funding.

## Author contribution

SMKA is the principal investigator and edits the manuscript, AT wrote the manuscript, LOR writing review and editing, and AKH runs the statistical analysis and methodology.

## Registration of research studies

1. Name of the registry:

2. Unique Identifying number or registration ID:

3. Hyperlink to your specific registration (must be publicly accessible and will be checked):

## Guarantor

Seyed Mohammad Kazem Aghamir.

## Consent

Patients enter the study after signing the written informed consent.

## Availability of data and material

Information, data, and photos will be provided if requested.

## Provenance and peer review

Not commissioned, externally peer-reviewed.

## Declaration of competing interest

There is no conflict of interest.
